# A novel chromatin regulator-related immune checkpoint related gene prognostic signature and potential candidate drugs for endometrial cancer patients

**DOI:** 10.1186/s41065-022-00253-w

**Published:** 2022-10-18

**Authors:** Zesi Liu, Hongxia Yang, Ziyu Chen, Chunli Jing

**Affiliations:** 1 Department of Gynecology and Obstetrics, The First Affiliated Hospital of Dalian Medical University, Dalian, 116000 Liaoning Province China; 2 Department of Gynecology and Obstetrics, The Second Affiliated Hospital of Dalian Medical University, Dalian, 116000 Liaoning Province China

**Keywords:** Chromatin regulators, Immune checkpoints, Endometrial cancer, TCGA, Prognosis

## Abstract

**Background:**

Endometrial cancer (EC) is the most common gynecologic malignancy in developed countries and its prevalence is increasing. As an emerging therapy with a promising efficacy, immunotherapy has been extensively applied in the treatment of solid tumors*.* In addition, chromatin regulators (CRs), as essential upstream regulators of epigenetics, play a significant role in tumorigenesis *and* cancer development.

**Methods:**

CRs and immune checkpoint-related genes (ICRGs) were obtained from the previous top research. The Genome Cancer Atlas (TCGA) was utilized to acquire the mRNA expression and clinical information of patients with EC. Correlation analysis was utilized for screen CRs-related ICRGs (CRRICRGs). By Cox regression and least absolute shrinkage and selection operator (LASSO) analysis, prognosis related CRRICRGs were screened out and risk model was constructed. The Kaplan–Meier curve was used to estimate the prognosis between high- and low-risk group. By comparing the IC50 value, the drugs sensitivity difference was explored. We obtained small molecule drugs for the treatment of UCEC patients based on CAMP dataset.

**Results:**

We successfully constructed a 9 CRRICRs-based prognostic signature for patients with UCEC and found the riskscore was an independent prognostic factor. The results of functional analysis suggested that CRRICRGs may be involved in immune processes associated with cancer. Immune characteristics analysis provided further evidence that the CRRICRGs-based model was correlated with immune cells infiltration and immune checkpoint. Eight small molecule drugs that may be effective for the treatment of UCEC patients were screened. Effective drugs identified by drug sensitivity profiling in high- and low-risk groups.

**Conclusion:**

In summary, our study provided novel insights into the function of CRRICRGs in UCEC. We also developed a reliable prognostic panel for the survival of patients with UCEC.

**Supplementary Information:**

The online version contains supplementary material available at 10.1186/s41065-022-00253-w.

## Introduction

In developed countries, uterine corpus endometrial carcinoma (UCEC) is the most common gynecologic malignancy, and its incidence is increasing [1]. In general, UCEC patients have a relatively good prognosis*,* but those with advanced UCEC have a poor treatment response, and therefore, their prognoses are worse [2].

A multiple signature model would fundamentally improve the prognostic value compared to a single biomarker, and multigene expression signatures have also been reported in various cancers for prognosis prediction [3, 4]. In this study, we aimed at constructing a signature based on chromatin regulators (CRs)-related immune checkpoint related genes (ICRGs) to predict overall survival (OS) in UCEC patients.

Epigenetics refer to the modifications of gene activity without changing the DNA sequence [5, 6]. During epigenetic remodeling, tumor cells acquire immunotolerance that provide them with the ability to escape the supervision of the body’s immune system [7]. It has been established that abnormal epigenetic changes can contribute to carcinogenesis [8, 9]. CRs are essential upstream regulators of epigenetics that regulate epigenetic alterations in three main ways: DNA methylation, histone modification, and chromatin remodeling [10, 11]. On the one hand, CRs can encode and decode various modifications on cytosines and histones, such as methylation and demethylation [12, 13]. On the other hand, chromatin remodelers can disrupt the association between the nucleosome and DNA and initiate a nucleosome repositioning, that can result in abnormal epigenetic modifications [10, 13, 14]. Previous studies have demonstrated that dysregulated expressions of CRs and the corresponding functional aberrations are associated with a variety of cancer-related biological processes that also affect UCEC such as inflammation [15], autophagy [16], proliferation [17], and apoptosis [18]. For example, HMGA1 is a chromatin remodeler that is thought to be effective in predicting prognostic outcomes in UCEC [19]. HMGA1 expression can be regulated by KIFC1, which is involved in the aerobic glycolysis of UCEC cells leading to proliferation of tumor cells. HMGA1 can also enhance the aggressiveness of tumor cells through the HMGA1-MMP-2 pathway [20]. CDK1 is also as a member of the CRs that promotes UCEC malignant progression through the PVT1/miR-612/CENP-H/CDK1 axis. Therapeutic inhibition of CDK1 activity can trigger apoptosis and cause a G2/M phase arrest of cell cycle [21], which can improve the prognostic outcome of UCEC patients.

Immune checkpoints (ICs) are immune regulators of stimulatory and suppressive pathways that play important roles in maintaining self-tolerance and regulating the type, extent, and duration of immune responses [22]. Malignant cells evade the immune system and change the tumor microenvironment through activating ICs [23, 24]. Previous studies have revealed that the unique immunological profile of UCEC makes it a promising target for immunotherapy [25]. A growing number of studies have demonstrated that immune checkpoint blockade (ICB) therapy which targets PD-1 and PD-L1 is effective in improving the prognosis of UCEC patients [26–28]. Clinical studies on ICB treatment for the remaining ICs, such as LAG3 [29] and TIGIT [30], have been conducted and yielded many results. In addition, other ICRGs, including agonists of stimulatory checkpoint pathways, inducible ICOS, CD40, or molecules targeting tumor microenvironment (TME) components, are coming into our attention [24].

Despite the striking clinical benefit of ICB therapy in multiple cancer types, the response rate of most patients to ICB therapy is less than 20–30% [31]. Therefore, combining with other types of anticancer therapies is considered as a novel strategy to enhance the therapeutic efficacy of ICB therapy [32]. Research has gradually revealed the role of CRs in regulating the expression of ICRGs and in antitumor immunity [33, 34]. For example, the findings from Lin et al. showed that loss of SETDB1-TRIM28 complex can upregulate CD247 expression and increase the infiltration of effector CD8 + T cells through activation of the cyclic GMP–AMP synthase (cGAS)–stimulator of interferon genes (STING) pathway and thus synergizes with ICB [35]. Indeed, it was also demonstrated by Di Zhao et al. They found that the knockdown of the chromatin remodeler, CHD, can remodel the TME by regulating the expression of ICGs in prostate cancer resulting in the reduction of the number of myeloid-derived suppressor cells (MDSC) in the TME and an increase in the number of CD8 + T cells. These effects significantly increase prostate cancer responsiveness to ICI leading to an improved patient prognosis [36]. Taken together, we argue that chromatin regulators-related immune checkpoint related genes (CRRICRGs) can also serve as potential biomarkers and therapeutic targets to improve the response rate of UCEC patients to ICB therapy. However, to our knowledge, no study has been conducted to investigate the relationship between CRs, ICRGs, and UCEC, and therefore, we are hoping to fill this gap. In this study, we investigated the expression levels of CRs in UCEC through bioinformatic analysis and screened 14 genes associated with prognosis in chromatin regulators-related immune checkpoint related genes (CRRICRGs). We successfully developed a multigenic prognostic panel based on 9 CRRICRGs and determined their role in tumor immunity in UCEC patients.

## Materials and methods

### Data collection and screening for CRs-related ICGs (CRRICGs)

Based on previous studies, we found 870 CRs [37] (Supplement Table 1) and 79 ICRGs [22, 38, 39] (Supplement Table 2), and obtained their mRNA expression in 547 UCEC tissues. We also obtained on 52 normal tissues from the Cancer Genome Atlas (TCGA, https://portal.gdc.cancer. gov) and the Gene Expression Omnibus (GEO) datasets (GSE63678 and GSE17025). Differential expression genes (DEGs) were searched for with the “limma” R package by comparing tumor tissues with normal tissues. We screened DEGs by a Wilcoxon signed rank test (false discovery rate, FDR < 0.05). The correlation analysis between CRs and ICRGs was performed using Spearman’s correlation. P values less than 0.05 were considered statistically significant.

**Table 1:** Gene list and coefficient

Gene symbol	Coefficient
BNTL9	0.0423
CD40LG	-0.3037
CD47	0.0107
HLA-DMB	-0.0072
HLA-DRB5	-0.0008
HLA-G	-0.0110
TNFRSF14	-0.0155
TNFRSF18	-0.0129
TNFRSF4	-0.0026

**Table 2:** The 8 small drugs of CMP dataset analyses results

CMAP names	*P*-value	*N*	Enrichment	Percent non-null
Tamibarotene	0.000068	4	-0.927	100%
Fluoride	0.000264	2	-0.911	100%
DL-Mevalonic acid	0.000369	2	-0.893	100%
Panobinostat	0.000504	2	-0.862	100%
Isoguanine	0.003157	2	-0.858	100%
Dinoprostone	0.003287	2	-0.831	100%
Vitamin D3	0.003521	2	-0.812	100%
Simvastatin	0.007725	2	-0.806	100%

### Construction and validation of a prognostic model based on CRRICRGs

Using the univariate Cox method, we firstly screened for 13 CRRICRGs associated with UCEC prognosis, and the *p*-value was corrected using the Benjamini & Hochberg (BH) correction approach. Considering the large number of predictor variables under study, we utilized the Least Absolute Shrinkage and Selection Operator (LASSO) method to select a subset of predictor variables that predict the outcome best while maintaining a good model fit by using the “glmnet” package. Ten-fold cross-validation was used to compute the optimal lambda shrinkage coefficient that minimizes cross-validated error and the largest value of lambda within one standard error of this optimal value. After obtaining the optimal lambda values, the variables with the best predictive power of the model were selected for prognostic model and risk score construction. [40]. Ultimately, we obtained the 9 CRRICRGs and their coefficients. Expression validation was performed by downloading 142 tissues expression data from the GTEx web portal (www.gtexportal.org). The risk score was calculated using the following risk score formula (1). The median risk score was used to divide patients into high- and low-risk groups. Survival analysis (the “survminer” package) was performed using Kaplan–Meier curve to evaluate the prognosis in two groups. The 1-, 3-, and 5-year ROC curves were drawn to assess the prognostic value of the signature using the “survival” and “TimeROC” packages. Then, the GSE119041 (*n* = 50) dataset downloaded from GEO were employed as an external validation set to confirm the prognostic value of the CRRICRGs-based signature.

1 $$\mathrm{risk}\;\mathrm{score}=\sum(\mathrm{expression}\;\mathrm{value}\;\mathrm{of}\;\mathrm{each}\;\mathrm{gene}\times\mathrm{and}\;\mathrm{its}\;\mathrm{coefficient})$$

### Developing a nomogram that incorporates clinical features based on risk score

The risk model’s and clinicopathological characteristics’ prognostic significances were further investigated using univariate and multivariate Cox analysis. Meanwhile, the relationship between the panel and clinicopathological characteristics was also analyzed. A nomogram was developed based on clinical variables and the CRRICRGs-based signature to provide reliable predictions of 1, 3, and 5-year survival of UCEC patients. Calibration curve analyses were also conducted to determine the suitability of our nomogram for clinical use.

### Functional enrichment analysis, Protein–protein Interaction (PPI) and Gene Set Enrichment Analysis (GSEA)

The STRING database (http://www.string-db.org/) and Cytoscape software were used to construct and visualize the PPI network. Gene Ontology (GO) and Kyoto Encyclopedia of Genes and Genomes (KEGG) pathway enrichment analyses were performed for the prediction of the potential functions of the 9 CRRICRGs through the “clusterProfiler” and “pathview” packages [41, 42]. GSEA was performed to investigate the underlying molecular mechanisms among low- and high-risk groups, a *p*-value < 0.05 and an FDR < 25% were considered statistically significant.

### Analysis of immune cell infiltration

Using TIMER, CIBERSORT, CIBERSORT-ABS, QUANTISEQ, MCPcounter, XCELL, and EPIC, we evaluated the infiltration level of immune cells between high-risk groups and low-risk groups. The correlation between the risk score and immune-cell characteristics in UCEC patients was explored through Spearman correlation analysis. Furthermore, the expressions of immune checkpoint genes were explored to predict the effect of immune checkpoint blockade (ICB) therapy [43].

### Screening for potential small molecule drugs and drug sensitivity analysis

To screen for potential drugs, based on CRRICRGs for reversing or inducing the biological states of UCEC, the CRRICRGs were inputted into the Connectivity MAP database (CMAP, https://portals.broadinsti tute.org/cmap/) [44]. Enrichment scores ranging from − 1 to 1 were analyzed and we thought that the drug with negative scores could be beneficial for UCEC treatment. The set threshold was with a *p*-value < 0.01, *n* ≥ 2, a percent non-null = 100, and an enrichment < -0.8. To evaluate the sensitivity difference of drugs between high- and low-risk group, the “pRRophetic” package was utilized to analyze the half-maximal inhibitory concentration (IC50) of anti-cancer drugs using the Genomics of Drug Sensitivity in Cancer (GDSC, http://www.cancerrxgene.org/) database. A *p*-value < 0.05 was considered statistically significant.

### Statistics analysis

**Fig. 1:**
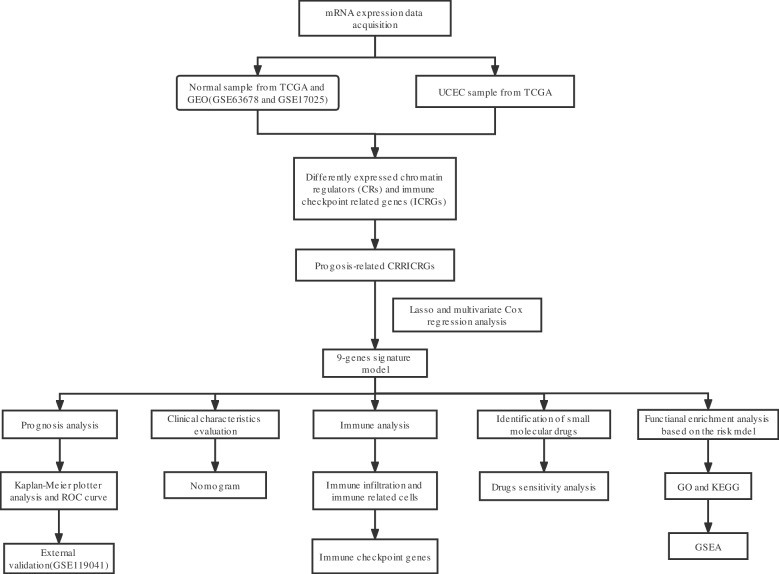
The flowchart of whole process of data analysis

All statistical analyses were conducted using R packages [R software (version 4.2.0)]. The differences between the two groups were compared by Wilcoxon signed-rank test. A *p*-value < 0.05 was considered statistically significant (*p*-value < 0.001 = *** , *p*-value < 0.01 = ** , and *p*-value < 0.05 = *). The process and study design are presented in a flow-chart (Fig. 1).

## Results

### Identification of prognosis-related differentially expressed CRRICRGs

**Fig. 2:**
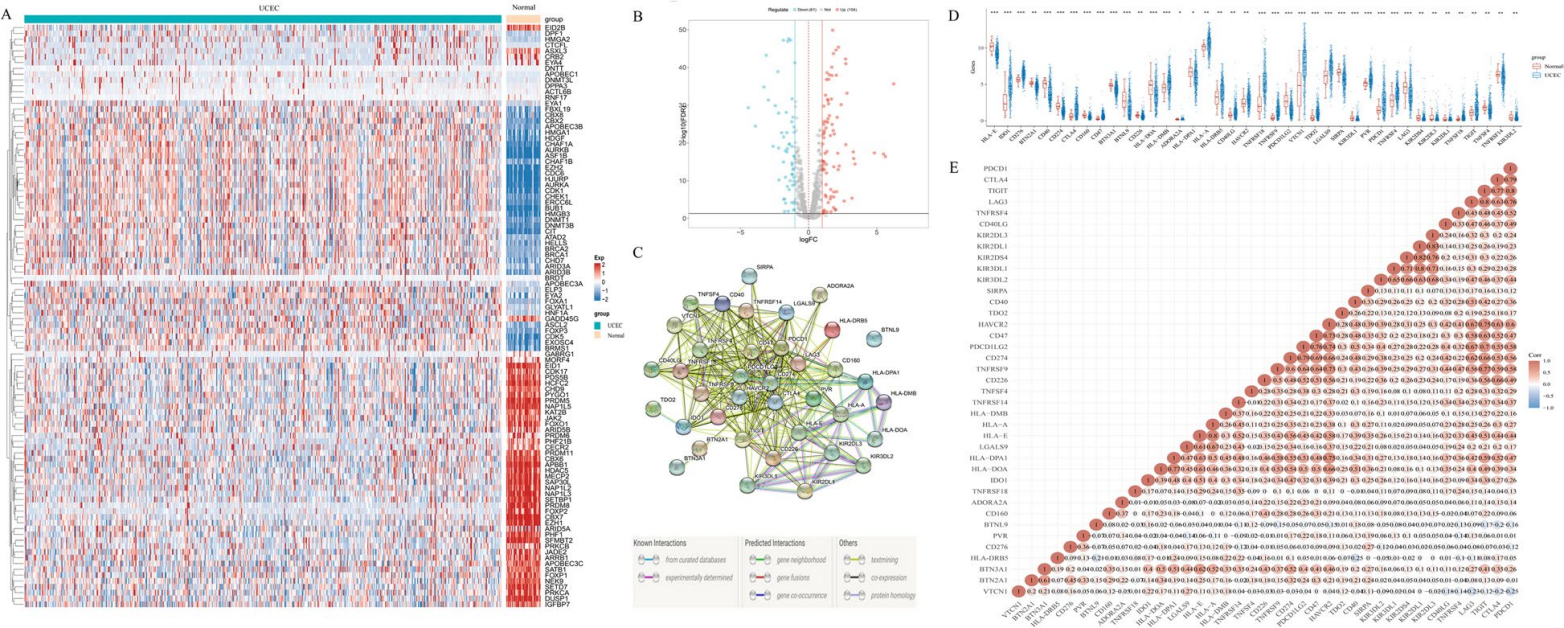
Gene expression and correlation. **A** Comparison of CRs expression profiles; **B** Volcano plots showing up-regulated and down-regulated CRs (104 up-regulated and 61 down regulated); **C** PPI network showing the intersection of 40 differentially express CRRICRGs; **D** CRRICRGs expression profiles; **E** The correlation of the 40 differentially express CRRICRGs

A total of 165 CRs were shown to be differentially expressed in UCEC tissues when compared with normal tissues (Fig. 2A). These included 104 upregulated genes and 61 downregulated genes (Fig. 2B). After correlation analysis of the differentially expressed CRs with ICRGs, 68 CRRICRGs were screened (Supplement table 3) and 40 out of 68 CRRICRGs were differentially expressed between UCEC tissues and normal tissues (Fig. 2D). 40 target genes were used to construct the PPI network (Fig. 2C) and the correlation among genes was shown in Fig. 2E. Using Cox regression analysis, we evaluated the prognostic value of these 40 differentially expressed CRRICRGs and obtained 13 prognosis-related CRRICRGs (Supplement Fig. 1A). The intersected genes were screened and extracted as shown in Veen diagram (Supplement Fig. 1B).

### Construction and validation of a multigene signature based on prognosis-related differentially expressed CRRICRGs

**Fig. 3:**
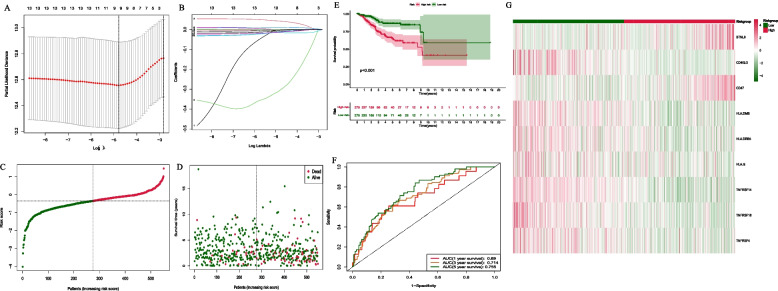
The development of a gene signature to predict patients OS. **A**, **B** Lasso-Cox regression analysis; **C** Risk scores of UCEC patients; **D** Survival status of UCEC patients; **E** Kaplan–Meier survival analysis of patients between high- and low-risk groups; **F** The 1-, 3- and 5-year ROC curve to predict the survival status; **G** Heatmap showed the differences of 9 genes based on riskscore

We constructed a signature containing 9 CRRICRGs (BTNL9, CD40LG, CD47, HLA-DMB, HLA-DRB5, HLA-G, TNFRSF14, TNFRSF18, TNFRSF4) using LASSO Cox regression analysis and demonstrated its ability to predict the prognosis of UCEC patients (Fig. 3A and B). Meanwhile, the relevant coefficients of 9 CRRICRs were obtained (Table 1), and the risk score was calculated as follow:

2 $$\mathrm{risk}\;\mathrm{score}=(0.0423\ast\mathrm{BTNL}9\;\mathrm{expression})+(-0.3037\ast\mathrm{CD}40\mathrm{LG}\;\mathrm{expression})+(0.0107\ast\mathrm{CD}47\;\mathrm{expression})+(-0.0072\ast\mathrm{HLA}-\mathrm{DMB}\;\mathrm{expression})+(-0.0008\ast\mathrm{HLA}-\mathrm{DRB}5\;\mathrm{expression})+(-0.0110\ast\mathrm{HLA}-\mathrm G\;\mathrm{expression})+(-0.0155\ast\mathrm{TNFRSF}14\;\mathrm{expression})+(-0.0129\ast\mathrm{TNFRSF}18\;\mathrm{expression})+(-0.0026\ast\mathrm{TNFRSF}4\;\mathrm{expression})$$

According to the median risk score, patients were divided into High- and Low-risk groups (Fig. 3C and D). We analyzed the expression differences of 9 prognosis-related CRRICRGs (Fig. 3G) between the two groups and compared the prognostic outcomes of patients in the two groups. The results showed that the prognostic outcomes of patients in the Low-risk group were significantly better than those in the High-risk group (*p* < 0.001) (Fig. 3E). Furthermore, the multigene signature based on the 9 prognosis-related differentially expressed CRRICRGs can predict the 1-, 3- and 5-year OS of UCEC patients and the prognostic accuracy of the 9 CRRICRGs-based signature was 0.69 at 1-year, 0.714 at 3-year and 0.755 at 5-year (Fig. 3F). The result of expression validation further supports our findings described above (Supplement Fig. 2). Then, as an external validation of our model, we analyzed GEO dataset GSE119041, which contains included 50 UCEC patients. The panel was also considered to be effective (Supplement Fig. 3).

### Prognostic value of the clinicopathological characteristics and relationship between the signature and clinical features

**Fig. 4:**
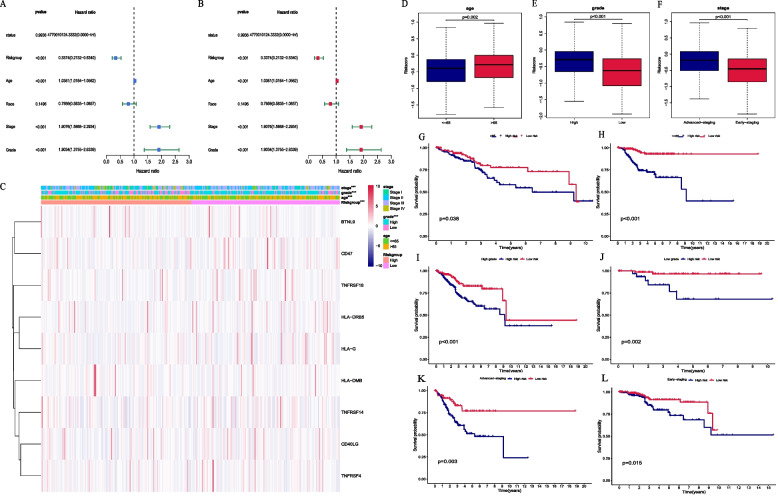
The relationship between riskscore and clinical characteristics. **A** Univariate Cox regression; **B** Multivariate Cox regression; **C** Heatmap demonstrating the association among the riskscore, the expression of 9 CRRICRGs and UCEC clinical characteristics including stage, grade and age; **D**-**F** The relationship between signature and clinicalpathological chracteristics. Patients in high -risk group were more frequently older with poorly differentiated (high-grade) tumors and advanced tumors stage; **G**-**L** Stratified analysis of survival of UCEC patients according to age, grade and stage

To assess the common clinicopathological characteristics and prognostic value of our prognostic panel, we sequentially performed univariate (Fig. 4A) and multivariate Cox analyses (Fig. 4B) and demonstrated that age (*p* < 0.001), stage (*p* < 0.001), grade (*p* < 0.001), and risk score (*p* < 0.001) independently predict the prognostic outcome of patients (Fig. 4C). Wilcoxon signed-rank test was utilized to explore the relationship between those clinicopathological characteristics and our multigene signature. The results suggested that patients in the low-risk group were younger (*p* = 0.002), and the tumors showed a greater frequency of a higher degree of differentiation (*p* < 0.001) and an earlier tumor stage (*p* < 0.001) (Fig. 4D-F). In addition, we hypothesized that the prognostic signature still has a prognostic value in subgroups based on clinicopathological characteristics [45]. Therefore, a stratification analysis was further conducted, and the results supported our hypothesis that CRRICRGs-based signature showed excellent performance in predicting outcome in age > 65 (*p* = 0.038), age <  = 65 (*p* < 0.001), high grade (*p* < 0.001), low grade (*p* = 0.002), advanced-staging (*p* = 0.003) and early-staging (*p* = 0.015) (Fig. 4G-L).

### Developing a nomogram that incorporates clinical characters

**Fig. 5:**
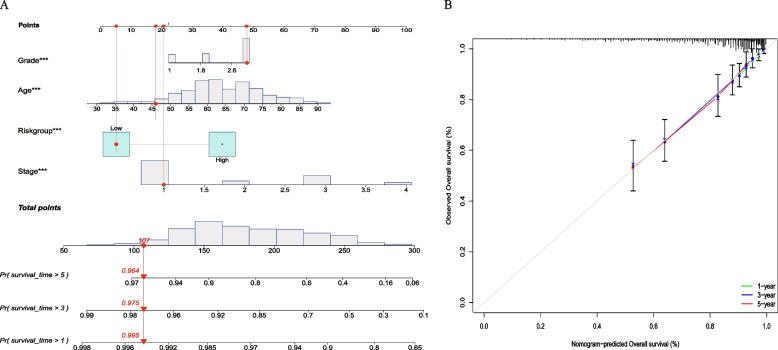
Construction of a predictive nomogram. **A** Nomogram to predict the 1-, 3- and 5-year survival of UCEC patients; **B** Calibration curve for the nomogram model

The above results suggest that the clinicopathological features and the CRRICRGs-based signature were associated with the prognostic outcome of UCEC patients. To graphically evaluate the survival probability of an individual, a nomogram that integrated clinical variables and prognostic signature was developed based on the TCGA-UCEC dataset to predict the 1-, 3- and 5-year survival time of UCEC patients (Fig. 5A). Furthermore, the calibration plot indicated that the nomogram operated in line with the ideal model (Fig. 5B).

### PPI and functional analyses between High-risk and Low-risk groups

**Fig. 6:**
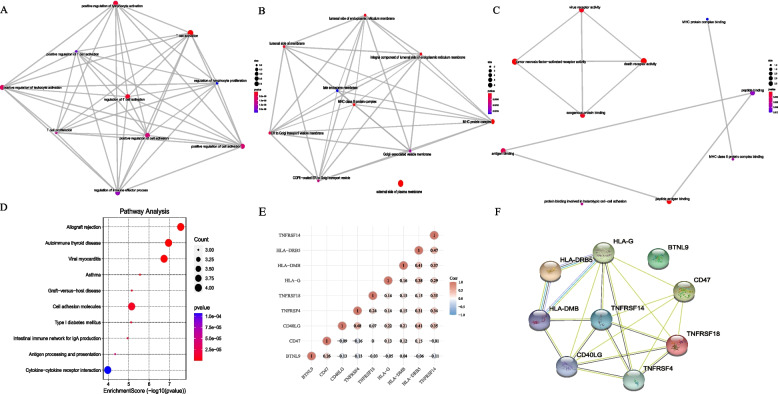
The functional enrichment analyses and the correlation of 9 CRRICRGs. **A** The emapplot from BP enriched pathway; **B** The emapplot from CC enriched pathway; **C** The emapplot from MF enriched pathway; **D** KEGG analysis; **E** The correlation of gene expression; **F** The PPI network of the prognostic 9 CRRICRGs

The correlation of 9 genes was shown in Fig. 6E. The PPI network of the prognostic 9 CRRICRGs was constructed using STRING database and suggested that HLA-G and CD40LG may be the hub genes (Fig. 6F). On the other hand, biological process (BP) analyses showed that T cell activation, positive regulation of lymphocyte activation, and positive regulation of leukocyte activation are the biological activities in which the 9 CRRICRGs are primarily involved (Fig. 6A). The result of cellular component (CC) analysis showed that the 9 CRRICRGs are involved in the composition of the external side of the plasma membrane, the integral component of the luminal side of the endoplasmic reticulum membrane, and MHC protein complex (Fig. 6B). Tumor necrosis factor-activated receptor activity, death receptor activity, and peptide antigen binding were mainly enriched according to the molecular function (MF) analysis (Fig. 6C). KEGG pathways analysis also indicated that CRRICRGs are enriched in several immune- and metabolism-related pathways (Fig. 6D). GSEA analysis was performed, and the enriched pathways in the high-risk group are presented in Supplement Fig. 4.

### Immune characteristics analysis

**Fig. 7:**
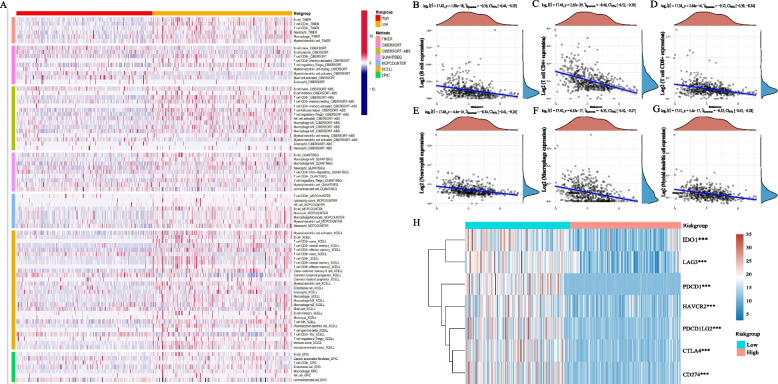
Immune characteristics analysis. **A** Immune cells infiltration between high-risk groups and low-risk groups. There was a larger proportion of immune components and more immune infiltration in low-risk group; **B** The relationship between riskscore and 6 immune cell subtypes. The expression of 6 main immune cells’ subtypes was negatively correlated with the RiskScore; **C** Expression difference of 7 key immune checkpoint genes between two groups. The low-risk group exhibited higher immune checkpoint expression levels

The analyses of TIMER, CIBERSORT, CIBERSORT-ABS, QUANTISEQ, MCPCOUNTER, XCELL, and EPIC were performed to explore the relationship between the panel and immune infiltration. Visually, most immune cells are significantly different between the high and low risk group and low-risk patients appeared to have a larger proportion of immune components and more immune infiltration (Fig. 7A). Next, we investigated the relationship between risk score and 6 main immune cells’ subtypes (B cell, CD4 + T cell, CD8 + T cell, Neutrophil, Macrophage and Mycloid dendritic cell) [46]. The results showed a negative correlation between risk score and the infiltration of six immune cells (Fig. 7B-G). ICB therapy has become an effective treatment for endothelial cancer [47]. Therefore, the correlation between the expression of 7 key ICs [48] and risk score was explored, and we found l ow-risk patients with UCEC had significantly higher expression of ICs than high-risk patients (Fig. 7H).

### Identification of small molecule drugs

By uploading nine CRRICRGs to the CMAP database, we obtained eight small molecule drugs that may be effective for the treatment of UCEC patients. These drugs were tamibarotene, fluoride, DL-Mevalonic acid, panobinostat, isoguanine, dinoprostone, vitamin D3, and simvastatin (Table 2).

### Drugs sensitivity analysis

By comparing the IC50 value of the common chemotherapeutic drugs based on the GDSC database, we found that patients in the high-risk group are more sensitive to cyclopamine, dimethyloxalylglycine (DMOG), dasatinib, and cytarabine (Supplement Fig. 5). Moreover, the IC50 value of docetaxel in the low-risk group of patients was higher than that in the high-risk group of patients, indicating that docetaxel is more effective in treating UCEC patients in the low-risk group (Supplement Fig. 5).

## Discussion

As ICB therapies targeting PD-1/PD-L1 [49] and CTLA-4 [50] have shown promising results in clinical trials, tumor immunotherapy is attracting more attention. Multigenic models that are based on ICRGs and with a high prognostic accuracy, have been constructed to predict OS and TEM in patients with a variety of tumors, including UCEC [51]. Previous studies not only found that CRs play an important role in carcinogenesis, but also revealed that they can regulate the body's anti-tumor immunity [52, 53]. The study by Zhu K et al. suggested that prognostic models that are constructed based on CRs, can predict OS and immune status of bladder cancer patients with a relative accuracy [54]. However, a CRRICRGs-based prognostic signature for UCEC has never been reported.

In this study, we screened 40 differentially expressed genes from 68 CRRICRGs using univariable Cox analysis of TCGA database and 13 CRRICGRs associated with the prognosis of UCEC. After LASSO-penalized Cox regression analysis, a prognostic signature containing 9 CRRICRGs (BTNL9, CD40LG, CD47, HLA-DMB, HLA-DRB5, HLA-G, TNFRSF14, TNFRSF18, and TNFRSF4), was constructed.

BTNL9 is a member of Butyrophilin (BTN) and Butyrophilin-like (BTNL) families, involved in inflammatory diseases and tumor development through the regulation of the T cell response [55, 56]. The activation of the RAS/MEK signaling pathway can downregulate BTNL9 expression [57], thereby deregulating BTNL9 inhibition of tumor cell invasion, leading to poorer prognostic outcomes [58]. Studies have identified a downregulation of BTNL9 expression in osteosarcoma [59], colon cancer [60], lung adenocarcinoma [61], and breast cancer [62]. Since CD40LG may trigger Th1-type immune responses, CD40LG can be used as a target for tumor therapy [63]. In addition, CD40LG can tightly regulate T cell activity by encoding CD40L, which can upregulate IL-2 expression by binding to CD40 on the surface of antigen-presenting cells (APCs) [64]. As a result, the CD40LG-CD40 axis can be used as a biomarker to predict various tumor prognosis [65]. Signal-regulatory protein α (SIRPα) is a CD47 ligand that is expressed on major APCs, including macrophages, dendritic cells, and neutrophils. SIRPα expression is upregulated in tumor cells and usually indicates worse prognosis [66, 67]. It was found that by blocking CD47 with anti-CD47 antibodies, the interaction between SIRPα and CD47 can be reduced, resulting in the enhancement of the ability of macrophages to present tumor cells [68]. As a tumor-associated gene, Human leukocyte antigen-DMB (HLA-DMB) is embedded in intracellular vesicles [69]. ERG silencing results in the downregulation of HLA-DMB expression, which reduces the release of class II-associated invariant chain peptide (CLIP). This would impair the ability of APC to present tumor cells to T cells [70]. Due to the involvement of HLA-DRB5 in the processing and presentation of inflammatory and immune-related antigens processing and presentation [71, 72], HLA-DRB5 may have a predictive value for survival rates of patients with malignant tumors [73]. HLA-G, located on chromosome 6 at region 6p21.3, plays an important role in maternal immune tolerance to the fetus [74]. Moreover, since HLA-G possesses immune blocking functions, this also suggests that HLA-G can be involved as an immune checkpoint in the development of many cancers [75]. However, it is interesting to note that in ovarian and rectal cancers, HLA-G expression is associated with a good prognosis [76, 77], suggesting that HLA-G is not only involved in the regulation of anti-tumor immunity, but its expression may also reflect genome integrity. The tumor necrosis factor receptor superfamily member (TNFRSF) 4, also known as OX40 or CD134, is usually expressed on the surface of CD4 + and CD8 + T cells [78]. Because TNFRSF4 can be involved in various immune responses through multiple pathways, including the induction of Th2 differentiation and the promotion of cytokine synthesis, it is often regarded as a specifical marker of T-cell activation [79, 80]. Encouragingly, recent evidence demonstrated that TNFRSF4 plays a key role in stabilizing TEM in UCEC, suggesting that TNFRSF4 may be a promising therapeutic target for T cell-mediated anti-tumor immunotherapy in UCEC patients [81–83]. TNFRSF14 is located on the short arm of chromosome 1q36 and can regulate a series of immune responses, including anti-tumor immunity, by encoding a type I transmembrane molecule [84]. TNFRSF14 activation can serve as a biomarker in evaluating various cancers’ prognosis, as it is closely associated with tumor growth and metastasis [85, 86]. However, in this study, the expression of TNFRSF14 was positively correlated with OS in patients with endothelial cancer and we speculated that this may be due to the following reasons: (1) TNFRSF14 activation can upregulate caspase-3 expression, and thus, promote the apoptosis of tumor cells [87]; and (2) TNFRSF14 can inhibit epithelial-to-mesenchymal transition (EMT) by blocking the PI3K-AKT signaling pathway through the inhibition of AKT expression [88]. TNFRSF18 plays a crucial role in modulating immune response and inflammation and is known as a reliable biomarker that can predict the prognosis of patients with endometrial cancer [89, 90].

The results of the Kaplan–Meier plotter analysis showed that patients in the low-risk group had a better prognosis compared with that in the high-risk group of patients. The AUC of ROC curves further illustrated the predictive value of our panel for OS in patients with UCEC. Using univariable and multivariable Cox analyses, we screened for 4 clinicopathological features (age, race, stage, and grade), associated with prognosis of endometrial cancer. Moreover, we also found that patients in the low-risk group were younger, and that the tumors have a greater frequency of a higher degree of differentiation and earlier tumor stage. To visualize the 1-, 3- and 5-year survival of endometrial cancer patients, we built a nomogram with an integrated risk score and clinical characters, and the calibration curve also showed that the nomogram has good predictive efficacy.

We next performed functional enrichment analysis on 9 CRRICRGs. The results of GO analysis suggested that regulation of immune cell activity, immune-related protein synthesis, and regulation of various receptor activities have the most frequent occurrences. The KEGG analysis indicated that CRRICRGs are mainly enriched in various immune- and metabolism-related pathways. The above results demonstrated that CRRICRGs can be involved in the development of UCEC through regulating multiple immune- and metabolism-related pathways and that they may also play important roles in regulating the TEM of UCEC. Meanwhile, the result of the analyses of TIMER, CIBERSORT, CIBERSORT-ABS, QUANTISEQ, MCPCOUNTER, XCELL, and EPIC indicated the relationship between risk score and immune infiltration. By analyzing the distribution of immune cells among the high- and low-risk groups, we found that the expression of APCs, including myeloid dendritic cell, B cells and macrophage, negatively correlate with risk score. This illustrated that patients in the low-risk group may have a greater number of APCs, which can present tumor cells to T cells, and thus, enhance anti-tumor immunity. This could partially explain why patients in the low-risk group had longer OS compared with that in the high-risk group of patients. In addition, the differential expression of 7 key ICs between the two groups is demonstrated in Fig. 7H. We found that the expression of ICs was significantly higher in the high-risk group of patients compared with that in the low-risk group of patients. Therefore, we thought that the high expression of ICs the high-risk group of patients may produce an immunosuppressive microenvironment that leads to a worse prognosis for patients. However, from another perspective, this finding also suggested that patients in the high-risk group are more responsive to ICB therapy. Finally, we screened 8 small molecular drugs that may be effective in the treatment of UCEC and found that cyclopamine, DMOG, dasatinib and cytarabine are effective drugs for treating patients in the high-risk group. Patients in the low-risk group might benefit from the treatments of Docetaxel.

This study has some limitations. All analyses were conducted and validated based on TCGA-UCEC, GEO and GTEx database, and further validation should be done using clinical samples in the future. In addition, more experiments are required to investigate the molecular mechanisms associated with CRRICRGs influence on UCEC progression.

## Conclusion

In summary, we identified 9 prognosis-associated CRRICRGs (BTNL9, CD40LG, CD47, HLA-DMB, HLA-DRB5, HLA-G, TNFRSF14, TNFRSF18, and TNFRSF4). A panel was developed, and we proved that it could predict the outcome and immune microenvironment in UCEC patients. Furthermore, our findings also suggested a potential therapeutic value of CRRICRGs for UCEC.

## Supplementary Information

**Additional file 1: Supplementtable 1.** The list of 870 chromatinregulators.

**Additional file 2: Supplementtable 2.** The list of 79 immunecheckpoint related genes.

**Additional file 3: Supplementtable 3.** The list of 68 CRRICRGs.

**Additional file 4: Supplement Figure 1.** 

**Additional file 5: Supplement Figure 2.** 

**Additional file 6: Supplement Figure 3.** 

**Additional file 7: Supplement Figure 4.** 

**Additional file 8: Supplement Figure 5.** 

## Data Availability

The datasets supporting the conclusions of this article are included within the article and are available from the corresponding author on reasonable request.

## Abbreviations

UCEC: Uterine corpus endometrial carcinoma
CRs: Chromatin regulators
ICRGs: Immune checkpoint related genes
OS: Overall survival
ICs: Immune checkpoints
ICB: Immune checkpoint blockade
TME: Tumor microenvironment
MDSC: Myeloid-derived suppressor cells
CRRICRGs: Chromatin regulators-related immune checkpoint related genes
PPI: Protein–protein interaction
GSEA: Gene set enrichment analysis
GO: Gene ontology
KEGG: Kyoto encyclopedia of genes and genomes
BP: Biological process
CC: Cellular component
MF: Molecular function
DMOG: Dimethyloxalylglycine
BTN: Butyrophilin
BTNL: Butyrophilin-like
APCs: Antigen-presenting cells
SIRPα: Signal-regulatory protein α
HLA: Human leukocyte antigen
CLIP: Class II-associated invariant chain peptide
TNFRSF: The tumor necrosis factor receptor superfamily member
EMT: Epithelial-to-mesenchymal transition
