# Supplementary figures and images for: A novel chromatin regulator-related immune checkpoint related gene prognostic signature and potential candidate drugs for endometrial cancer patients
Source: Hereditas. 2022 Oct 18;159:40. doi: 10.1186/s41065-022-00253-w (PMC9578220; doi:10.1186/s41065-022-00253-w)

A

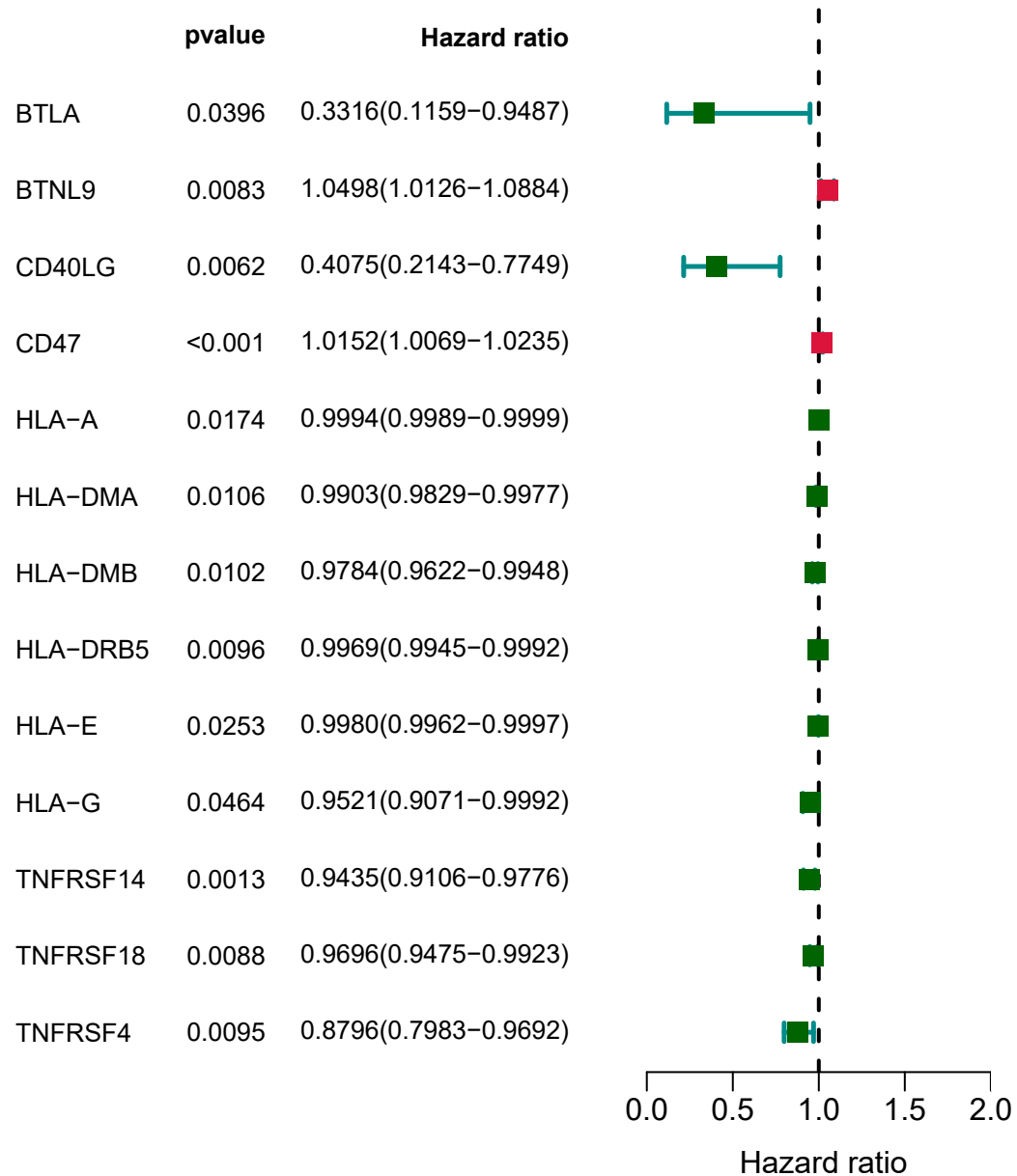

B

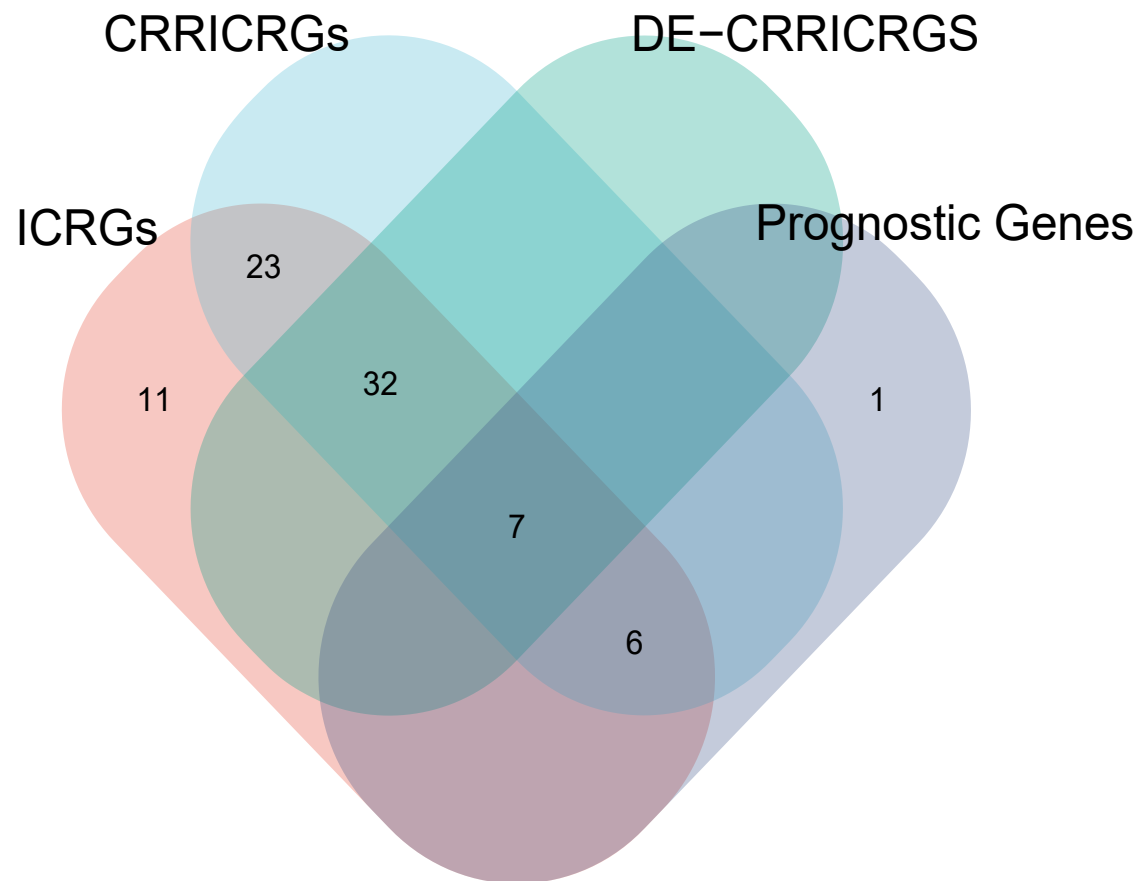

Supplement: Supplementary file 4 — Additional file 4: Supplement Figure 1. [file 41065_2022_253_MOESM4_ESM.pdf]

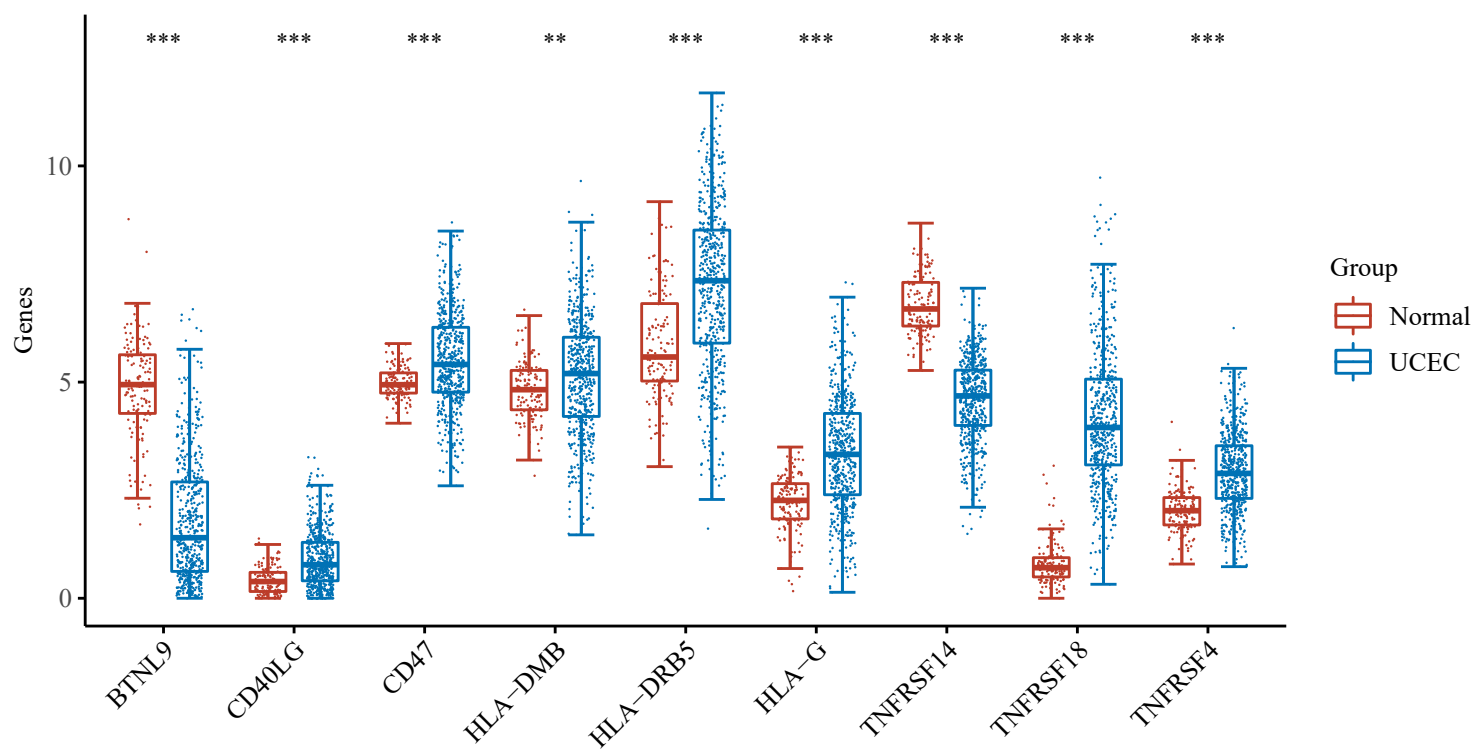

Supplement: Supplementary file 5 — Additional file 5: Supplement Figure 2. [file 41065_2022_253_MOESM5_ESM.pdf]

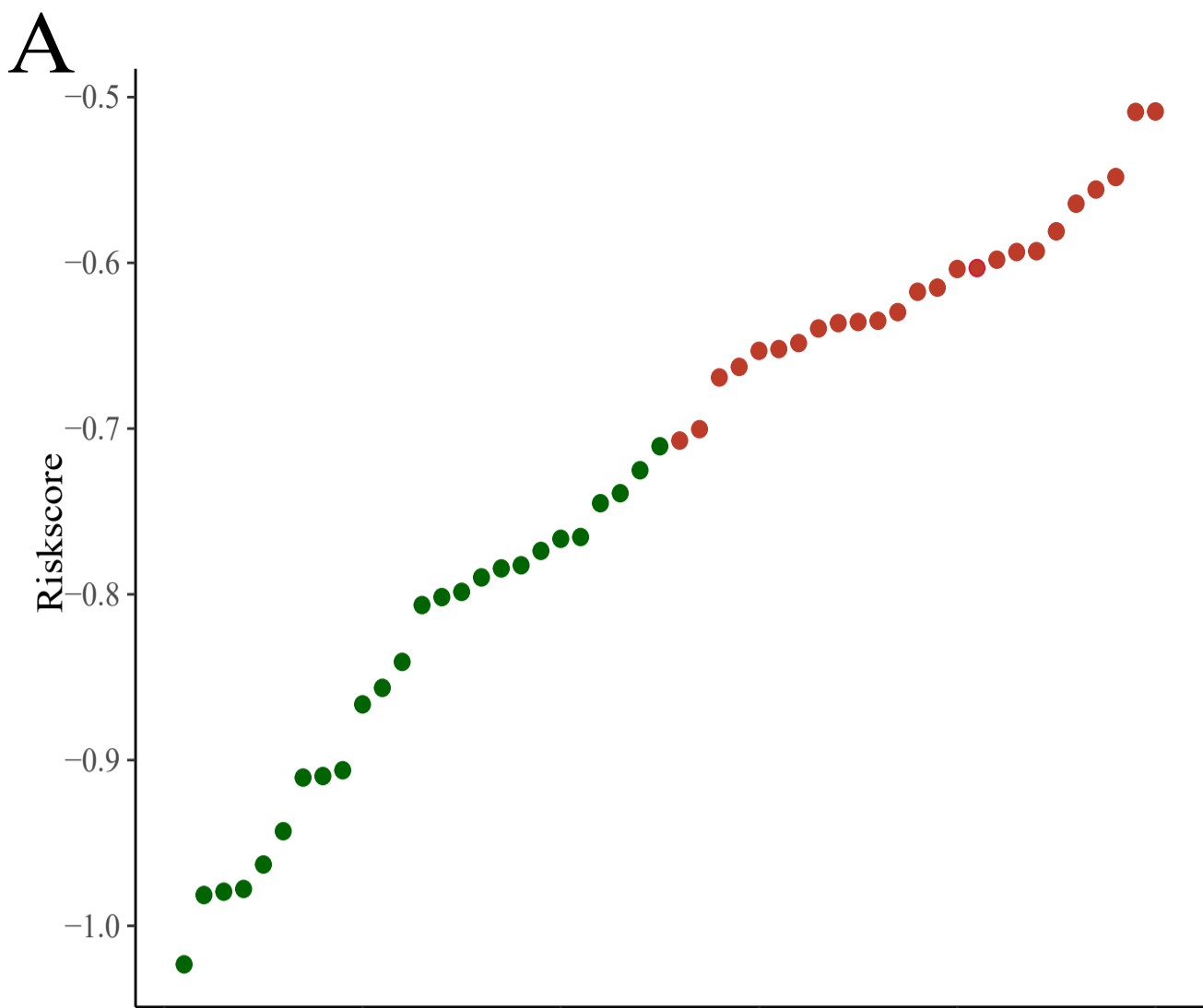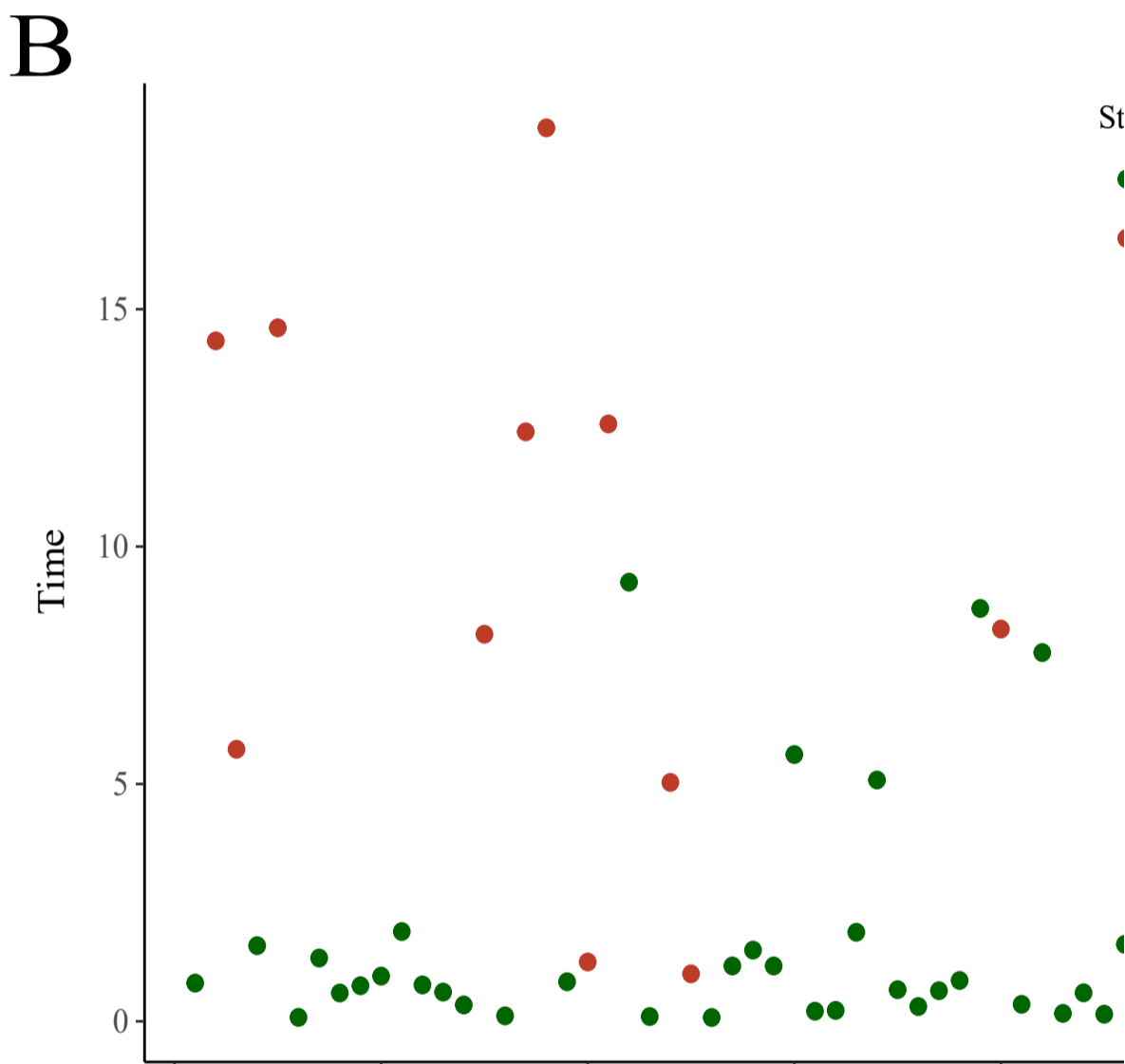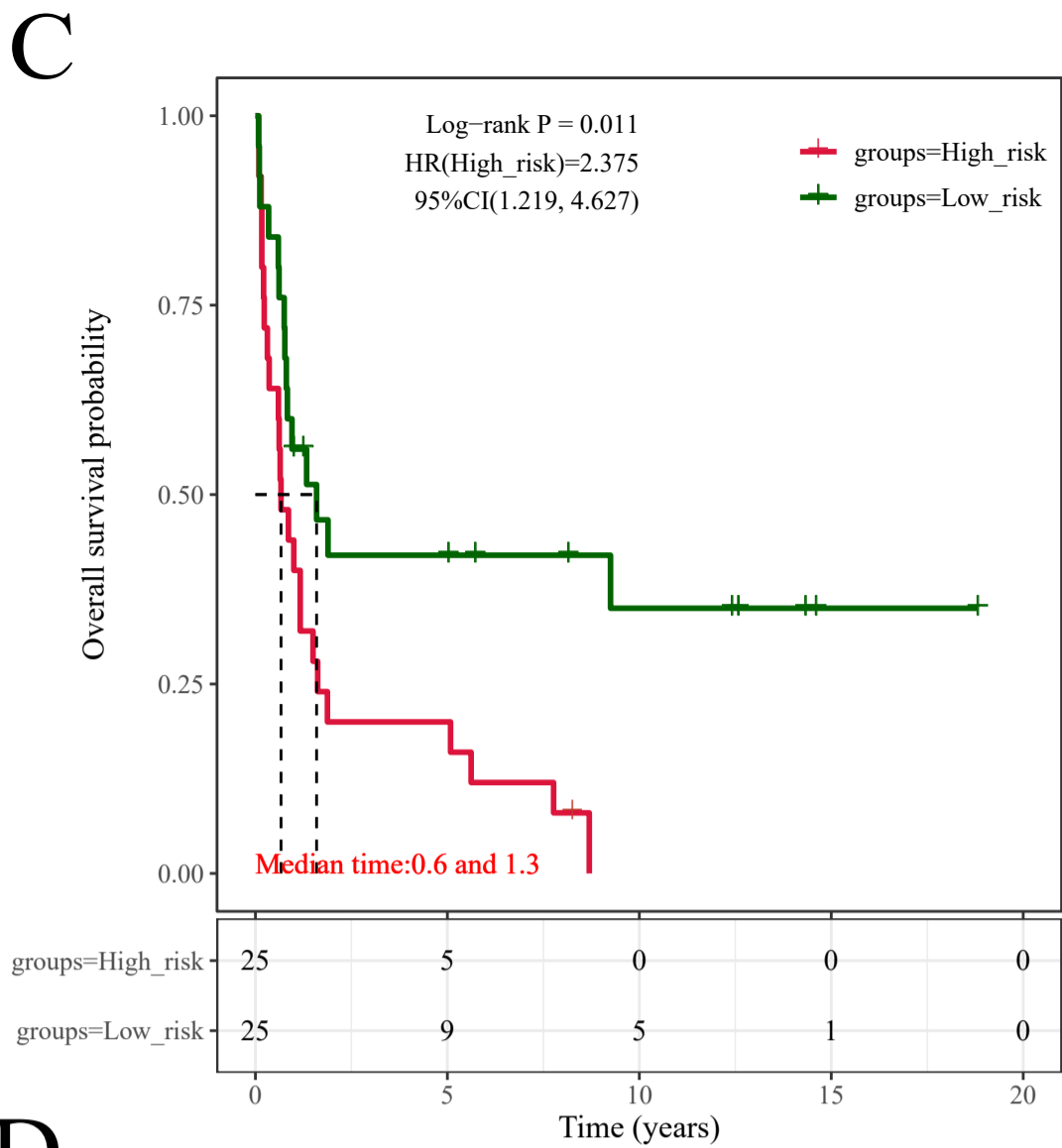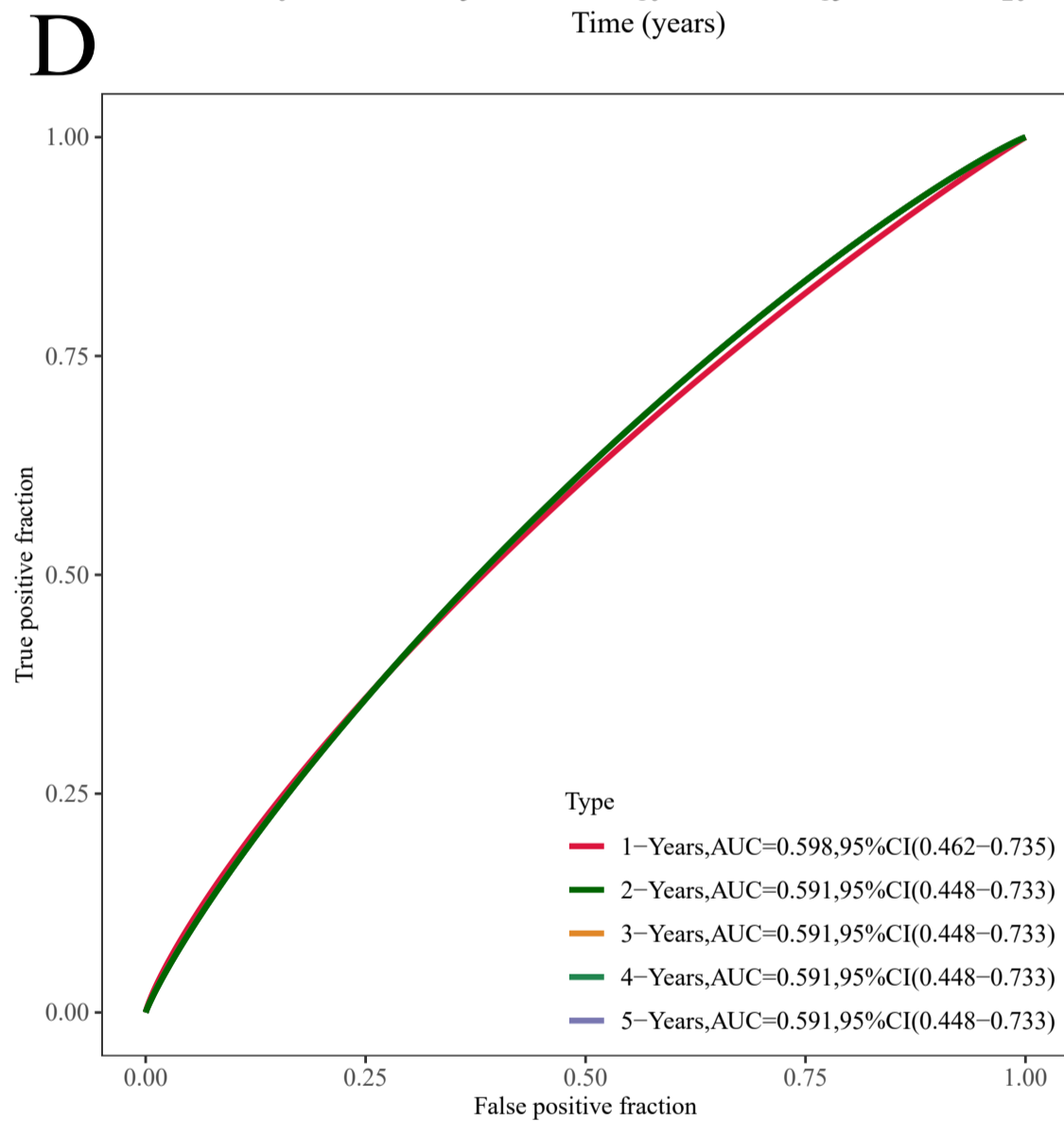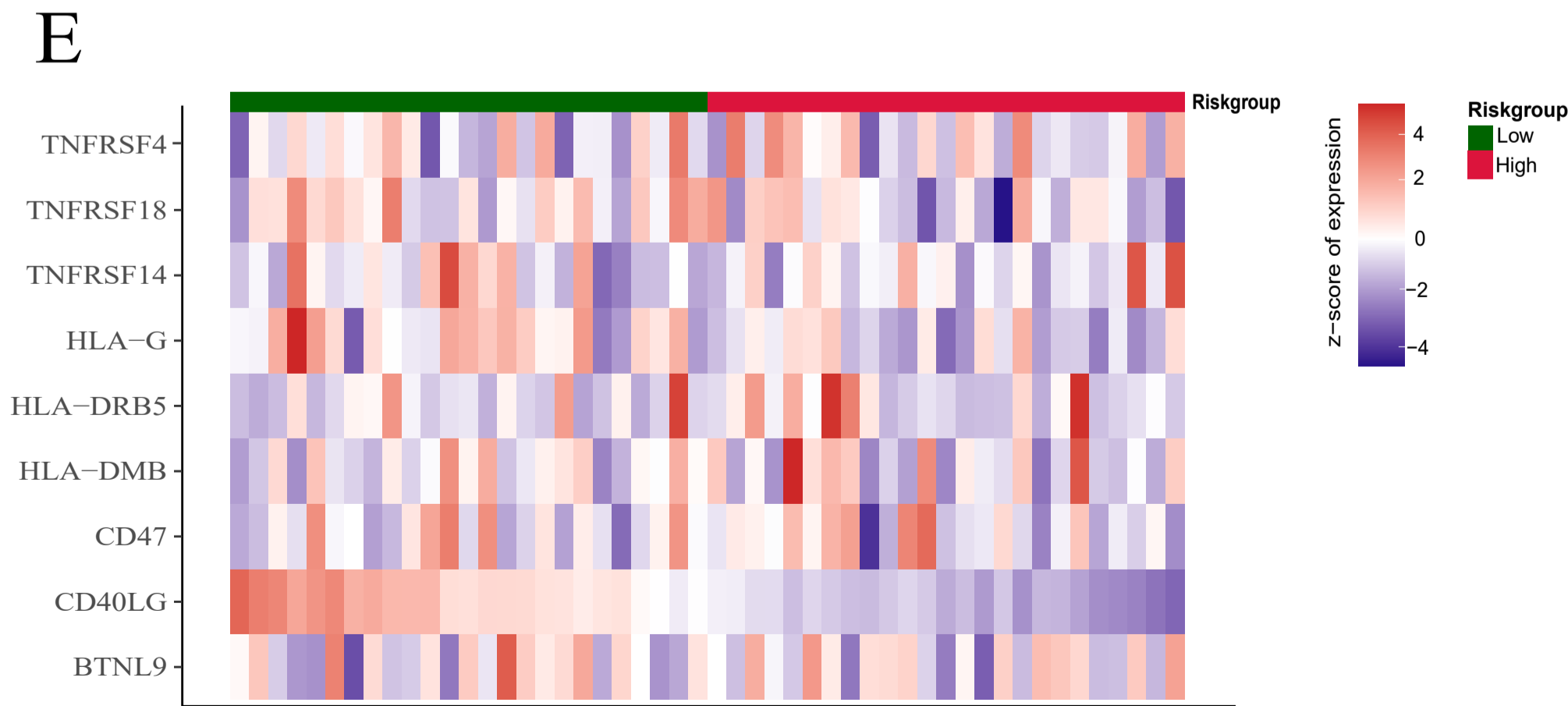

Supplement: Supplementary file 6 — Additional file 6: Supplement Figure 3. [file 41065_2022_253_MOESM6_ESM.pdf]

Running Enrichment Score

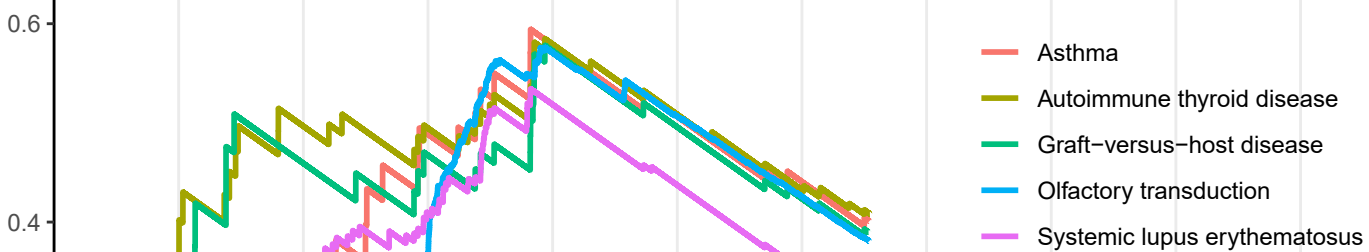

Ranked List Metric

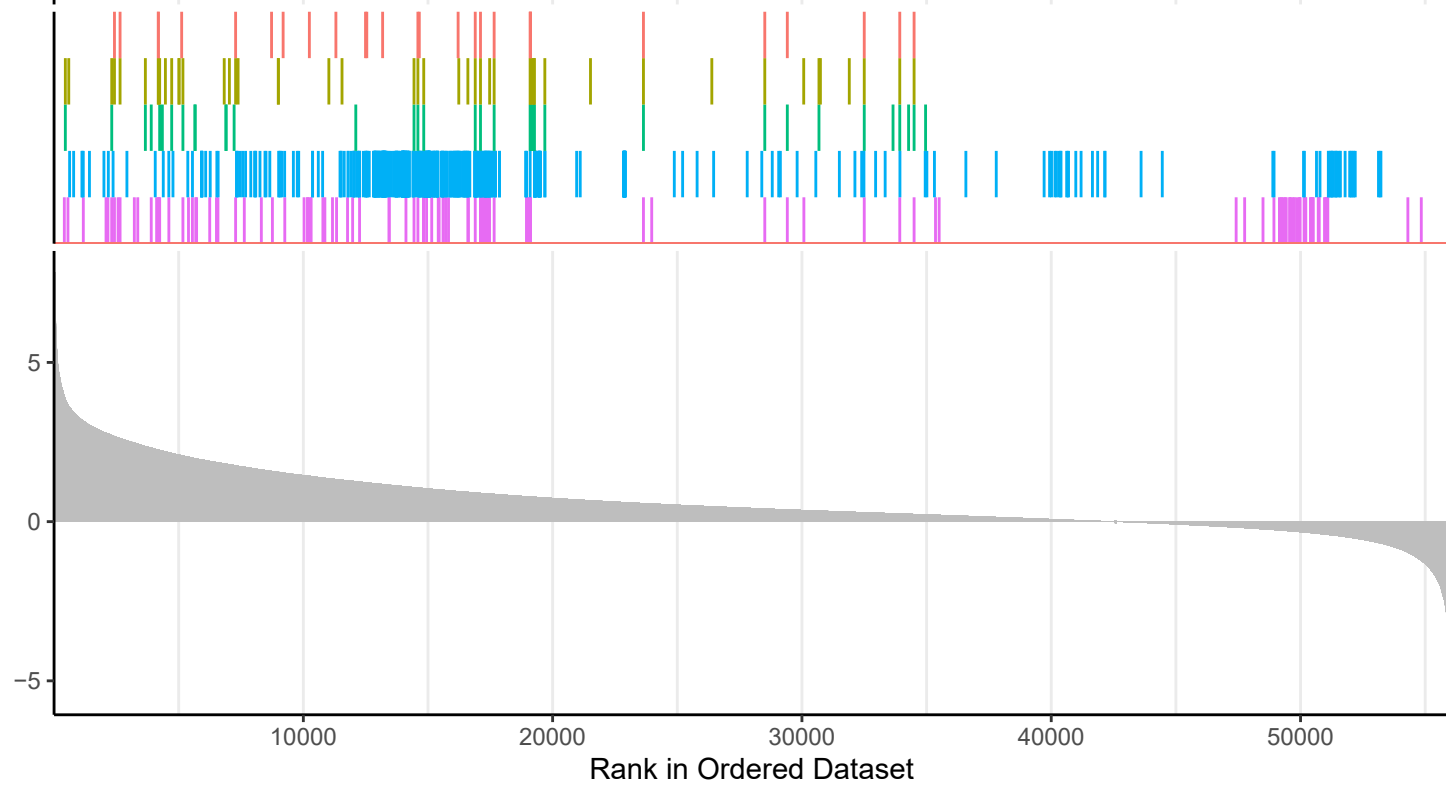

Supplement: Supplementary file 7 — Additional file 7: Supplement Figure 4. [file 41065_2022_253_MOESM7_ESM.pdf]

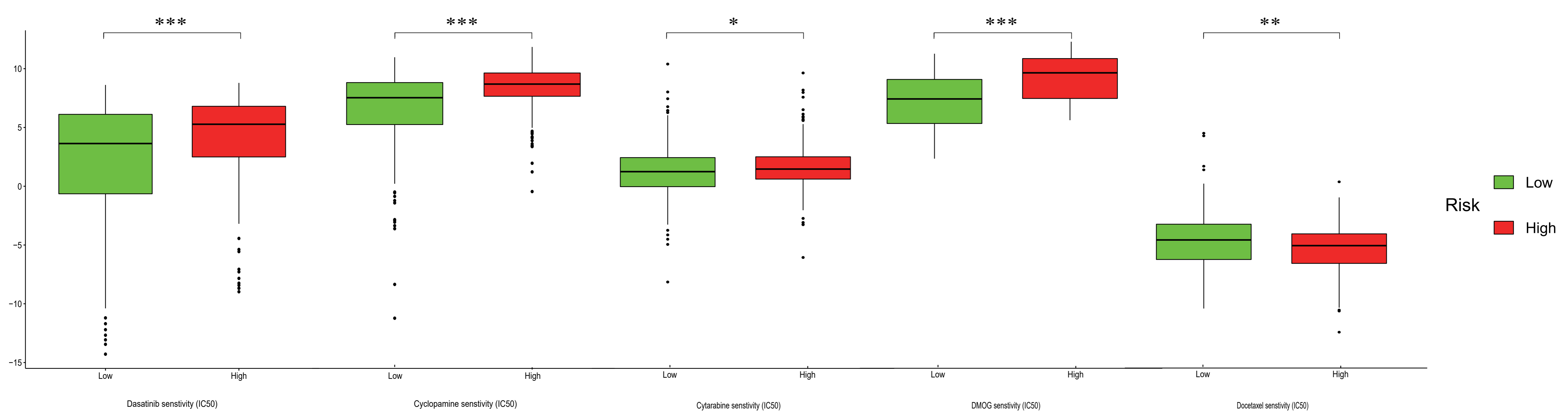

Supplement: Supplementary file 8 — Additional file 8: Supplement Figure 5. [file 41065_2022_253_MOESM8_ESM.pdf]
